# Transcriptome Proffling, Physiological and Biochemical Analyses Reveal Comprehensive Insights into Cadmium Stress in *Myricaria laxiflora*

**DOI:** 10.3390/plants13233433

**Published:** 2024-12-06

**Authors:** Yang Peng, Yu-Bing Yang, Jing-Cheng Wang, Mao-Yuan Tian, Xing-Hai Yuan, Zhi-Jiang Yang, You-Wei Zuo, Hong-Ping Deng

**Affiliations:** 1 Key Laboratory of Eco-Environment in the Three Gorges Reservoir Region, Ministry of Education, School of Life Sciences, Southwest University, Beibei, Chongqing 400715, China; 15182375576@139.com (Y.P.); yubing_yang2022@163.com (Y.-B.Y.); m13224042763@163.com (J.-C.W.); yzj18337179126@163.com (Z.-J.Y.); youweiz@swu.edu.cn (Y.-W.Z.); 2 Jiangjin Forestry Bureau, Chongqing 402260, China; 13883396399@163.com (M.-Y.T.); jjlyyxh_2020@163.com (X.-H.Y.)

**Keywords:** *M. laxiflora*, cadmium stress, morphological response, physiological and biochemical response, differentially expressed genes

## Abstract

With the expansion of cities and the development of industries, heavy metal pollution has caused a serious negative impact on the growth and development of animals and plants, which has become a global economic and social problem. Cadmium (Cd) is one of the main heavy metals that threaten the growth and development of plants, and it can lead to the imminent extinction of plants in severe cases. The part of upper reaches of the Yangtze River in China from Yibin to the Three Gorges Reservoir has been contaminated with varying degrees of Cd, and a rare and endangered plant called *Myricaria laxiflora* also lives in this area. The stress of heavy metal Cd on *M. laxiflora* populations is still unknown. In this study, we used the seedlings of *M. laxiflora* as materials, and adopted conventional physiological and biochemical analyses to characterize the morphological and physiological responses of *M. laxiflora* under different concentrations of Cd, and analyzed its response to Cd stress at the transcriptional level. The results showed that the wild population of *M. laxiflora* was stressed by the heavy metal Cd. High concentrations of Cd can inhibit the growth of *M. laxiflora. M. laxiflora* responded to the Cd stress through resistance substances such as malondialdehyde (MDA), hydrogen peroxide (H_2_O_2_), superoxide dismutase (SOD), catalase (CAT), and phytohormones such as auxin (IAA), gibberellin (GA) and abscisic acid (ABA). Transcriptome analysis was carried out on *M. lasiflora* seedlings exposed to 24 h, 48 h, and 72 h of Cd stress. Compared with 0 h (control), 2470, 11,707, and 11,733 differential expressed genes (DEGs) were identified, respectively. Among them, the number of down-regulated genes is more than the number of up-regulated genes. Transcriptome analysis showed that the upregulated genes were mainly enriched in MAPK signaling pathway, ethylene-induced pathway, ABA response pathway and other pathways, and the downregulated genes were mainly enriched in photosynthesis related pathways. Cd stress affected photosynthesis of *M. laxiflora*, and *M. laxiflora* may activate the MAPK signaling pathway through ethylene and ABA to improve the ability of Cd stress tolerance. These results reveal morphological changes, physiological and biochemical reactions and related key response pathways of *M. laxiflora* during Cd stress. It can provide a reference basis for habitat restoration and selection of wildlife environments for *M. laxiflora*.

## 1. Introduction

*M. laxiflora* belongs to the genus *Myricaria* of Tamaricaceae, which is classified as a national second-class key protected wild plant. It is a good tree species for beach soil stabilization and is also an ideal ornamental plant for gardening, and it also has certain medicinal value [1]. It is mainly distributed in the area of upper and middle reaches of the Yangtze River, and its population is small in Chongqing City.

With accelerated urbanization and industrialization, the problem of heavy metal pollution has become particularly prominent [2]. Among these pollutants, Cd is of particular concern because it poses an urgent and serious threat in the form of a divalent cation (Cd^2+^) [3]. Chemical speciation modeling of the rhizosphere solutions revealed that nearly all of the Cd was dissolved and distributed among the bioavailable Cd^2+^, Cl-complexed, and humates-complexed pools, with only small quantities of Cd adsorbed to K/Na-aluminosilicates. The slightly acidic pH range of 5.4–6.2 and the complexation with Cl^−^ and humates in the rhizosphere promoted the solubility of Cd and facilitated its transfer to plants [4]. In addition, the accumulation of Cd in plants can lead to damage to chloroplast structures [5]. This can reduce the viability of plants and lead to the risk of extinction. Therefore, elucidating the response mode of heavy metal Cd stress and the regulatory pathways behind it to reduce the damage of heavy metal stress on plants is an urgent issue nowadays [6].

In recent years, due to increased industrialization and urbanization, a certain degree of heavy metal pollution exists in the receding zone of the upper Yangtze River from Yibin to the Three Gorges reservoir section [7,8,9,10,11]. As a representative species of the upper Yangtze River from Yibin to the Three Gorges Reservoir in China, there is a lack of information on the physiological and biochemical response patterns and the key response pathways behind the heavy metal stress in *M. laxiflora.*

Excessive accumulation of Cd leads to excessive accumulation of reactive oxygen species (ROS), which significantly reduces the photosynthetic capacity of plants. Consequently, this leads to an increase in lipid oxidation, which inhibits plant growth and development. The activity of antioxidant enzymes has been recognized as a reliable indicator of plant response to Cd stress. These enzymes, such as SOD, POD and CAT, are effective in reducing the presence of ROS in plants [12]. In addition, common non-enzymatic antioxidants include glutathione (GSH) and ascorbic acid. GSH is known to attenuate heavy metal toxicity and activate gene expression [13]. Ascorbic acid scavenges polyunsaturated fatty acid (PUFA) radicals produced during membrane lipid peroxidation [14]. Proline (Pro) has been shown to reduce lipid peroxidation and free radical levels, thereby maintaining the integrity of plant cell membranes exposed to Cd stress [15]. Adsorption, translocation, and accumulation of heavy metals in plant cells usually depend on the following processes: binding and trapping to the root cell wall, transport into the cytoplasm, and sequestration in leaf vesicles [3]. These processes are tightly regulated by a range of proteins and families of transporter protein genes [16]. Recently, several studies have shown that some of the transporter protein genes of the *NRAMP* (Natural Resistance-Associated Macrophage Protein Genes) family are positively associated with heavy metal uptake in rice, barley, and transgenic *Arabidopsis* [16,17]. In addition, a significant decrease in Cd accumulation in roots, branches, and seeds was observed after the knockdown of NRAMP5 in rice. It is widely accepted that abiotic stresses, such as Cd stress, trigger the synthesis of many transcription factors that regulate different signaling pathways in plants and responses to these stresses. WRKYs are a group of transcription factors in plants that have been shown to play a crucial role in regulating many biotic and abiotic stresses. Recent studies in various plants have shown that WRKYs can enhance Cd tolerance by controlling the expression of downstream target genes [18]. In *Arabidopsis*, *WRKY13* can positively regulate Cd tolerance by activating the expression level of *PDR8* [19], and another study by Jia et al. showed that *TaWRKY70* in wheat can positively regulate *TaCAT5* to enhance Cd tolerance in transgenic *Arabidopsis* [20]. In addition, it was shown that heavy metal stress led to an increase in abscisic acid (ABA) and ethylene (ETH) concentrations and a decrease in auxin (IAA) concentrations. This suggests that genes associated with endogenous hormones are responsive to heavy metal stress [21].

Therefore, the aim of this study was to investigate the mode of response to Cd stress and possible key response pathways in sparsely flowered water hyacinth branches. Specifically, the main objectives of this study were to (1) reveal the heavy metal stress in wild populations of *M. laxiflora*; (2) determine the morphological, physiological, and biochemical responses of *M. laxiflora* to Cd stress; and (3) evaluate the key response pathways of *M. laxiflora* to Cd stress. In order to solve these problems, we investigated the morphological, physiological, and transcriptional responses of *M. laxiflora* under Cd stress at different concentrations and heavy metal contents in the soil of *M. laxiflora’s* habitat.

## 2. Result

### 2.1. Analysis of Heavy Metal Pollution Status in M. laxiflora Habitat Soil

The wild populations of *M. laxiflora* were stressed by the heavy metal Cd. The average contents of Cu, Zn, Cd, and Pb in the soil of the wild population were 228.90 ± 11.32 mg/kg, 112.78 ± 27.85 mg/kg, 0.45 ± 0.19 mg/kg, and 42.29 ± 14.48 mg/kg, respectively (Table 1). Among these, the pollution level of Cd was considered relatively high as moderate pollution, Pb was classified as mild pollution, and it was not contaminated by Cu and Zn (Table S1).

### 2.2. Changes in Cd Content and Morphological Characteristics of M. laxiflora Seedlings Under Cd Stress

**Figure 1:**
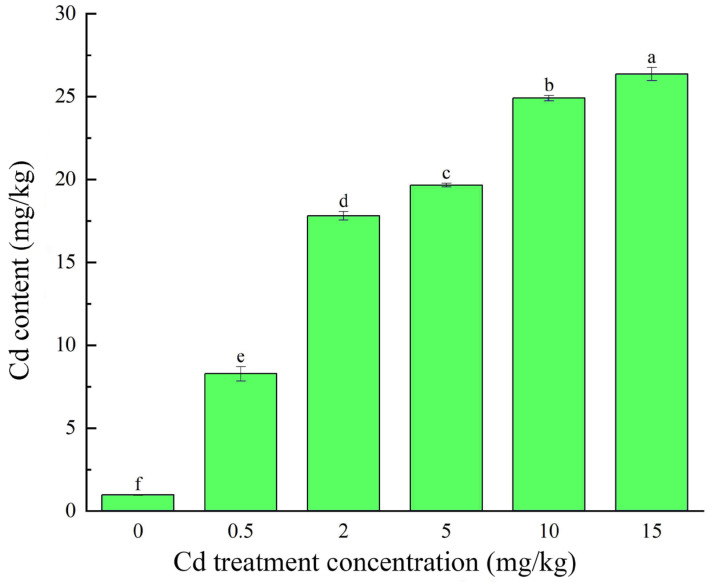
Changes in Cd content in seedlings of *M. laxiflora* under Cd stress. Note: different letters indicate significant differences in indicators between treatments (*p* < 0.05). Plant samples: seedlings from soil culture for 90 d. Number of biological replicates: 3.

The Cd content in the leaves of the seedlings of *M. laxiflora* showed an increasing trend with the increase in Cd stress concentration, and there was a significant difference (*p* < 0.05) between all groups (Figure 1).

A high concentration of Cd stress inhibited the growth of *M. laxiflora.* The low concentration of Cd did not have significant effects on the plant height, root length, and fresh weight, but the high concentrations of Cd (5 mg/kg, 10 mg/kg, and 15 mg/kg) significantly inhibited its growth (*p* < 0.05) (Table 2). With the increase in Cd stress concentration, the growth of primary branches of *M. laxiflora* seedlings showed a decreasing trend while the growth of secondary branches decreased significantly (*p* < 0.05) (Table 3). The anatomical structure of the leaves of the seedlings showed that the upper and lower epidermis of the leaves under all treatments were structurally intact; the palisade cells were more developed and tightly arranged, but were loosely arranged compared with the control group after the Cd treatment concentration of 2 mg/kg; the spongy tissues were more loosely arranged compared with the control group after the treatment concentration of 0.5 mg/kg; and the xylem and phloem were tightly arranged with no significant changes among treatment groups (Figure S1). There was no significant change between treatment groups (Figure S1). Morphological characteristics of *M. laxiflora* under different concentrations of Cd stress showed no significant effects on low concentrations and significant inhibitions on high concentrations, which indicated that excessive concentration of Cd stress would inhibit the growth of *M. laxiflora.*

### 2.3. Characteristics of Physiological and Biochemical Changes in Seedlings of M. laxiflora Under Cd Stress

**Figure 2:**
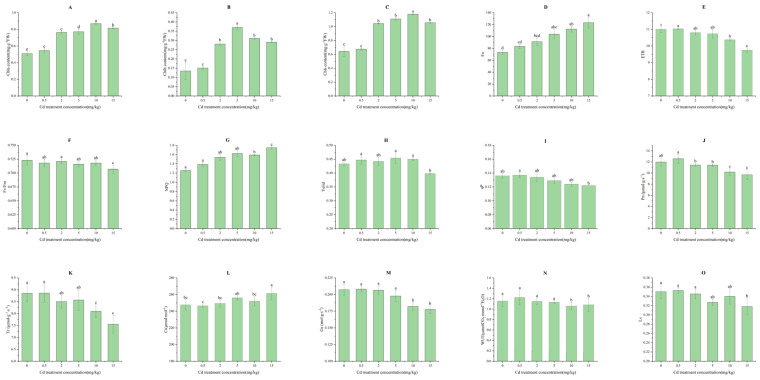
Photosynthetic response of *M. laxiflora* to Cd stress. (**A**–**C**): chlorophyll response; (**D**–**I**): response to chlorophyll fluorescence; (**J**–**O**): gas exchange. Plant samples: seedlings from soil culture for 90 d. Number of biological replicates: 3; Different letters indicate significant differences in indicators between different treatments (*p* < 0.05).

The chlorophyll a(chla), chlorophyll b(chlb), and chlorophyll contents of *M. laxiflora* increased with the increase in Cd stress concentration, but when the Cd stress concentration exceeded 10 mg/kg, chla, chlb, and chlorophyll contents of *M. laxiflora* began to show a decreasing trend (Figure 2A–C).

Fluorescence origin (Fo) and nonphotochemical quenching (NPQ) showed an increasing trend with the increase in Cd stress concentration. The levels of Fo reached a significant difference (*p* < 0.05) when the Cd stress concentration was 5 mg/kg, and NPQ reached a significant difference (*p* < 0.05) when the Cd stress concentration was 2 mg/kg. Maximal photochemical efficiency of PSII in the dark (Fv/Fm), photochemical quenching (qP), and electron transport within photosynthetic (ETR) showed an increasing trend with the increase in Cd stress concentration. Cd treatment concentration decreased and reached significant difference levels (*p* < 0.05) at Cd stress concentrations of 15 mg/kg, 15 mg/kg, and 10 mg/kg, respectively, indicating that Cd stress disrupted photosystem II of *M. laxiflora* and inhibited its electron transport in sparse-flowered *M. laxiflora* branch seedlings. The actual photochemical efficiency of PSII in the light (Yeild) showed an upward and then downward trend with the increase in Cd treatment concentration, which indicated that Cd stress could inhibit electron transport of *M. laxiflora*, although it did not have a significant effect on the primary light energy capture in *M. laxiflora* (Figure 2D–I).

The net photosynthesis (Pn), stomatal conductance (Gs), transpiration rate (Tr), and stomatal limitations (Ls) of *M. laxiflora* decreased under high Cd stress. The levels of Pn, Gs, and Tr reached a significant difference (*p* < 0.05) when the Cd stress concentration was 10 mg/kg. The levels of Ls reached a significant difference (*p* < 0.05) when the Cd stress concentration was 2 mg/kg. The water utilization rate (WUE) increased and then decreased, reaching significant levels at 10 mg/kg and 15 mg/kg (*p* < 0.05). Intercellular carbon dioxide (Ci) increased and reached a significantly different level (*p* < 0.05) when the Cd stress concentration was 2 mg/kg. It indicated that non-stomatal factors were the dominant factors that led to the decrease in the net photosynthetic rate. The reason might be that the high concentration of Cd damaged the photosystem II of *M. laxiflora* and inhibited electron transport, which led to the decrease in net photosynthetic rate (Figure 2J–O).

### 2.4. Characteristics of Biochemical Changes in Seedlings of M. laxiflora Under Cd Stress

**Figure 3:**
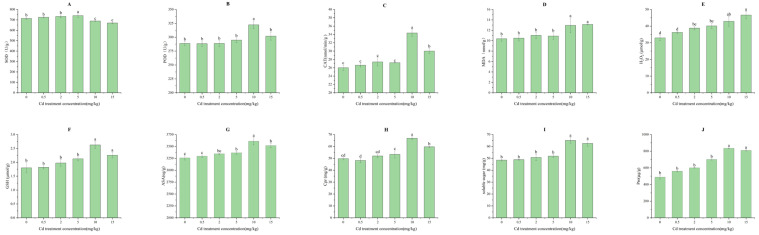
Antioxidant and osmoregulatory responses of *M. laxiflora* to Cd stress. (**A**–**E**): antioxidant response; (**F**–**J**): osmoregulatory response. Plant samples: seedlings from soil culture for 90 d. Number of biological replicates: 3; Different letters indicate significant differences in indicators between different treatments (*p* < 0.05).

The contents of malondialdehyde (MDA) and hydrogen peroxide (H_2_O_2_) tended to increase with the addition of Cd treatment concentration and were significantly higher than the control group at 10 mg/kg and 2 mg/kg, respectively (*p* < 0.05). It showed that Cd could easily cause lipid peroxidation in the cell membranes of *M. laxiflora* and impair the normal metabolism function of the cells. The activities of POD, SOD and CAT showed an increasing and then decreasing trend with the increase in Cd stress concentration, with the highest and significantly higher activities than the control at 10 mg/kg, 5 mg/kg and 10 mg/kg, respectively (*p* < 0.05), This suggested that low concentrations of Cd could enhance the antioxidant activity and the production of non-enzymatic antioxidants and antioxidant modulators of *M. axiflora* in order to enhance its scavenging ability against harmful substances such as oxygen free radicals. However, under high Cd stress, the stress response of Cd-induced seedlings was reduced (Figure 3A–E).

Similarly, the contents of soluble sugars, soluble proteins, free proline, GSH, and ASA in the seedlings showed an overall trend of increasing and then decreasing with the increase in Cd stress concentration. The contents of them reached the highest at 10 mg/kg of Cd stress concentration and all of them were significantly higher than the control group’s. (*p* < 0.05) (Figure 3F–J).

### 2.5. Changes in Endogenous Hormones in M. laxiflora Seedlings

**Figure 4:**
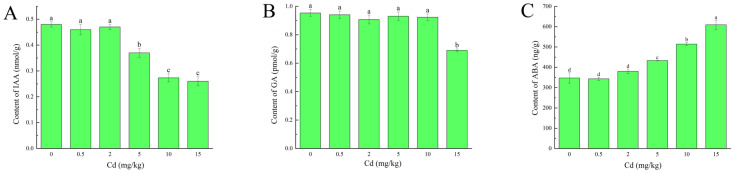
Effect of Cd stress on contents of endogenous hormones of *M. laxiflora.* (**A**) Changes in the content of IAA under cadmium stress; (**B**) Changes in the content of GA under cadmium stress; (**C**) Changes in the content of ABA under cadmium stress; Plant samples: fresh tissues from seedlings cultured in soil for 90 d. Number of biological replicates: 3; Different letters indicate significant differences in indicators between different treatments (*p* < 0.05).

The contents of IAA and GA in the seedlings showed a decreasing trend with the addition of Cd stress concentration (Figure 4A,B), and, respectively, reached significantly different levels with Cd stress concentration of 5 mg/kg and 15 mg/kg (*p* < 0.05). However, the content of ABA tended to increase with increasing Cd stress concentration (Figure 4C) and reached a significant level (*p* < 0.05) at 5 mg/kg. On the contrary, the content of ABA tended to increase with increasing Cd stress concentration (Figure 4C) and reached the level of significant difference (*p* < 0.05) at the Cd stress concentration of 5 mg/kg. Since ABA plays an important role in plant resistance to abiotic stresses, it is hypothesized that *M. laxiflora* may improve its tolerance to Cd stress by elevating the ABA content.

### 2.6. Analysis of Differentially Expressed Genes in Response to Cd Stress in M. laxiflora

The Cd content in the see dlings treated with 150 µmol/L Cd^2+^ increased with the treatment time, and the differences between the treatment groups were significant (*p* < 0.05) with the treatment of 24 h, 48 h, and 72 h. (The 0 h treatment group was the control group). Their Cd contents were significantly increased (*p* < 0.05) compared with the control group, which was 7.59, 8.80, and 12.44 times higher than those of the control group, respectively (Figure S2).

**Figure 5:**
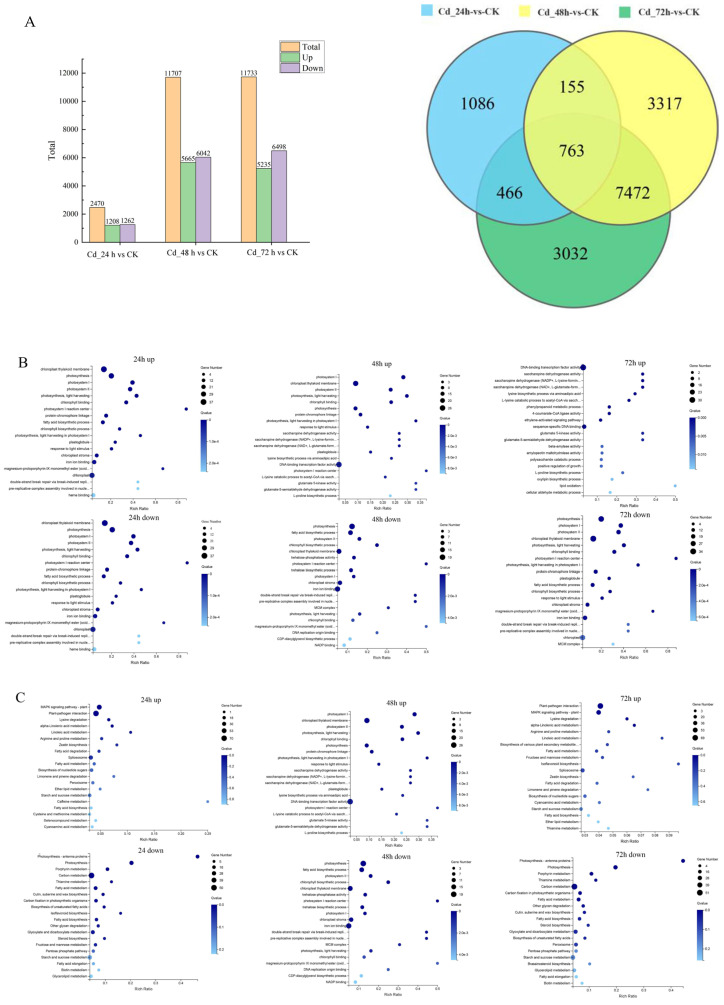
Genetic response of *M. laxiflora* to Cd stress. (**A**): Differential gene counts and differential gene Venn diagrams for Cd-stressed *M. laxiflora.* (**B**): Statistical chart of enriched GO terms of DEGs in *M. laxiflora* under Cd (top 20). (**C**): Statistical chart of KEGG pathways of differently expressed genes in *M. laxiflora* under Cd.

Compared with the control, 2470, 11,707, and 11,733 differential expressed genes (DEGs) were identified under 24 h, 48 h, and 72 h treatments, respectively, including 1208, 5565, and 5235 significantly up-regulated genes and 1262, 6042 and 6498 significantly down-regulated genes, respectively (Figure 5A). Along with the time of Cd stress extending, the number of genes involved in the stress response in *M. laxiflora* showed an additional trend, and the number of down-regulated genes was more than the number of up-regulated genes, which showed that Cd stress affected the gene expression of *M. laxiflora.*

GO enrichment analysis revealed that the expression levels of genes involved in photosynthesis and light harvesting, as well as photosystem II pathways were significantly down-regulated after 24 h, 48 h, and 72 h treatment, indicating that Cd stress affected the photosynthesis of *M. laxiflora* seedlings (Figure 5B).

KEGG pathway enrichment analysis revealed that the MAPK signaling pathway was upregulated and significantly enriched at 24 h, 48 h, and 72 h. The *MAPK* signaling pathway was upregulated and significantly enriched at 24 h, 48 h, and 72 h (Figure 5C).

**Figure 6:**
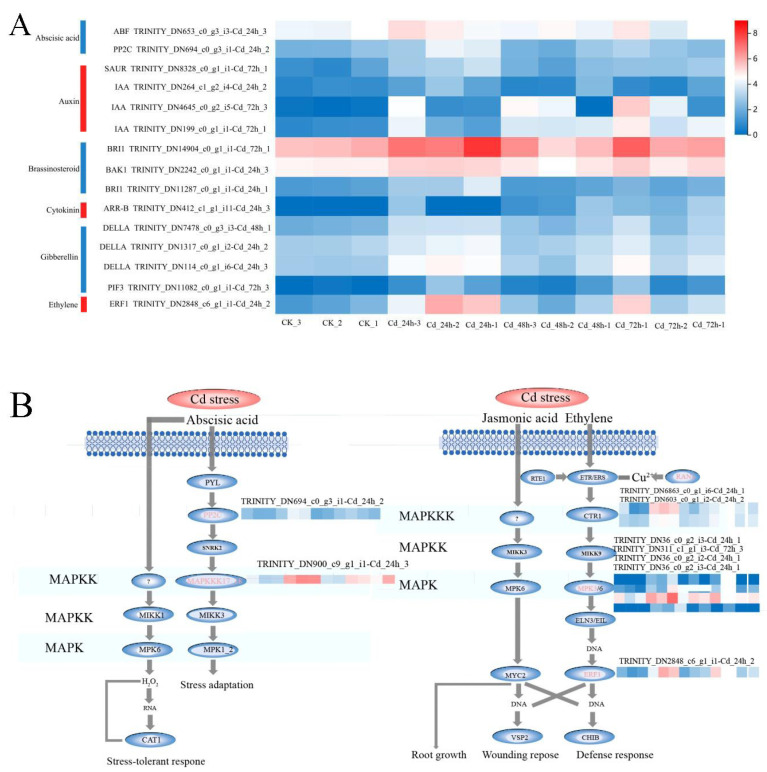
Analysis of phytohormone signaling and MAPK signaling pathways in *M. laxiflora* under Cd stress. (**A**) Heat map of phytohormone signaling in DEGs of *M. laxiflora* under Cd stress; (**B**) Predictive map of phytohormones involved in the MAPK signaling pathway in *M. laxiflora* under Cd stress; Abscisic acid, auxin, brassinosteroid, cytokinin, glibberellin, ethylene.

Given the critical role of the MAPK signaling pathway in conferring tolerance to abiotic stresses, including low temperature and heavy metal exposure, further heat map analysis showed that the expression levels of key genes *RAN1*, *MPK3*, and *ERF1* in the ethylene response pathway were significantly up-regulated under 24, 48, and 72 h of Cd stress. Therefore, it can be inferred that ethylene may activate the MAPK signaling pathway to enhance Cd tolerance in *M. laxiflora* (Figure 6).

## 3. Discussion

### 3.1. Cd Stress Affects the Growth and Development of M. laxiflora

Heavy metal pollution is a major environmental stress affecting plant growth and development. Heavy metal Cd inhibits various physiological processes in plants, including seed germination and seedling growth, photosynthesis, and antioxidant responses [22]. Therefore, in order to address the issue of Cd as a contaminant, it is crucial to understand the specific response of plants under Cd stress [23]. Previous studies have shown that morphological changes in plants can reflect the plant's response to the external environment. For example, changes in the morphology of leaves can reflect the response of plants to the environment [24]. For most plants, heavy metal stress at low concentrations does not significantly affect plant growth. However, when the concentration of heavy metals exceeds the tolerance range of the plant, it can lead to inhibition of plant growth and development [25,26]. Here, we show that the growth and development of *M. laxiflora* are significantly inhibited when subjected to high Cd stress concentrations. For example, the growth of secondary meristems of *M. laxiflora* was significantly reduced with increasing Cd stress concentration. This is similar to the results of Cd stress on *Sophora tonkinensis* [27]. In addition, the arrangement of spongy and fenestrated tissues of leaves under high Cd stress became loose, which was similar to the results of previous studies [28]. This suggests that the morphology of *M. laxiflora* changes in response to the environment when they are stressed by Cd, which is instructive for finding out whether *M. laxiflora* is stressed by Cd.

### 3.2. Cd Stress Affects the Physiological Functions of M. laxiflora

The chlorophyll of leaves is an important indicator to reflect the strength of leaf photosynthesis. When the concentration of heavy metal stress is too high, the structure of chlorophyll in plants will be damaged or the content will be decreased [29], thus affecting the photosynthesis of plants, which ultimately leads to the reduction in plant biomass [30]. In this study, the chlorophyll a, chlorophyll b, and total chlorophyll contents of *M. laxiflora* showed a trend of increasing and then decreasing with increasing Cd stress concentration, which was similar to the results of the studies on mulberry and sassafras under Cd stress [31,32], indicating that low concentrations of Cd promote chlorophyll synthesis. However, high concentrations of Cd inhibit chlorophyll synthesis, possibly due to the inhibition of enzymes related to chlorophyll synthesis or the production of oxidizing substances that damage the chloroplast structure, but also due to the replacement of Mg^2+^ in chlorophyll by Cd^2+^, which disrupts the structure of chlorophyll and leads to a decrease in chlorophyll content [33]. Chlorophyll fluorescence rapidly responds to the effects of adversity on photosynthesis, and it can reveal the intrinsic light energy utilization mechanism under adversity conditions [34,35]. The initial fluorescence yield can reflect the degree of permanent damage to PSII in plant leaves by adversity stress [36,37]. Maximum photochemical conversion efficiency, photochemical quenching coefficient, and non-photochemical bursting coefficient are closely related to PS II reaction centers [38,39,40]. In our study, we found that after 90 d of Cd stress, the initial fluorescence yield (Fo) tended to increase with the increase in Cd stress concentration, whereas the maximum photochemical efficiency was the opposite, which might be due to irreversible damage or reversible inactivation of PSII reaction center in plant leaves [41]. It was also found that the proper concentration of Cd contributed to the capture efficiency of primary light energy by PSⅡ in *M. laxiflora*, which was suppressed at high concentrations. This may be due to the fact that low concentrations of Cd stimulate chlorophyll synthesis and promote light energy uptake, whereas at high concentrations, the light reaction center is destroyed, leading to a decrease in maximum light quantum yield and electron transfer activity [42]. Heavy metal stress often leads to an increase in ROS content and a disruption of redox balance in plants [43]. The accumulation of ROS in plants leads to the production of membrane lipid peroxidation products represented by MDA, which can be toxic to plants. However, the plant body facing the accumulation of ROS is usually scavenged using enzymes such as SOD, CAT, and POD and substances such as antioxidants such as GSH, ASA, etc. [44]. In this experiment, it was found that the activities of three antioxidant enzymes, POD, SOD, and CAT, were promoted at low concentrations of Cd and inhibited at high concentrations, which is similar to the results of *Sophora tonkinensis* under Cd stress [27]. This suggests that low concentrations of Cd can increase the activity of antioxidant enzymes in response to Cd stress, but high concentrations of Cd can disrupt cellular functions and reduce the stress response capacity of the antioxidant system [45]. It was also found that the MDA content of *M. laxiflora* tended to increase with increasing Cd concentration, which was similar to the findings of Cd-stressed *Pogostemon cablin* [46]. This suggests that higher concentrations of Cd exacerbate membrane lipidation in *M. laxiflora* seedlings [43]. H_2_O_2_ content was also significantly increased under high Cd stress, suggesting that high concentrations of Cd resulted in the accumulation of ROS in *M. laxiflora* seedlings, similar to the results of a related study [47]. Osmoregulatory substances can maintain the intracellular osmotic potential and ensure the normal supply of water under heavy metal stress, thus ensuring the normal physiological function of cells. They play an important role in coping with heavy metal stress in addition to protecting enzymes required for cell metabolism [48]. In this study, we found that low concentrations of Cd could promote soluble protein production, soluble sugar synthesis, and proline synthesis in *M. laxiflora* seedlings, suggesting that in the low concentration range, *M. laxiflora* seedlings could resist Cd stress by increasing the contents of soluble proteins, soluble sugars, and free proline, but too high a concentration of Cd would disrupt the plant’s cellular membrane system and metabolic homeostasis, resulting in the reduction in its stress capacity [49]. It was also found that the production of GSH and synthesis of ASA were promoted when the Cd concentration was low, but the content of GSH and ASA decreased when the concentration of Cd treatment was too high, suggesting that the seedlings of *M. laxiflora* mitigated the toxicity of the heavy metal by increasing the content of GSH and ASA after being stressed by Cd, whereas the synthesis of GSH was limited by the high concentration of Cd stress [50,51]. Plant hormones play important roles in adversity and can regulate plant growth and development [52], among which IAA can effectively alleviate Cd-induced H_2_O_2_ accumulation [37]. GA belongs to the hormones that promote plant growth, which can reduce endogenous NO content and inhibit the synthesis of Cd transporter proteins, thus reducing the uptake of Cd and alleviating the toxicity of Cd [49]. The results showed that the content of IAA showed a decreasing trend with the increase in Cd stress concentration, which was similar to the results of Cd stress in *Cinnamomum camphora*, probably due to the intensification of membrane lipid peroxidation and the enhancement of the activity of indole acetic acid oxidase under the high concentration of Cd stress, which accelerated the decomposition of IAA [37]. ABA belongs to the group of hormones that inhibit plant growth and enhance plant resistance hormone, which plays an important role in Cd stress and can alleviate the toxicity of Cd to plants by regulating the antioxidant system [53,54]. With the increase in Cd stress concentration, ABA in *M. laxiflora* seedlings showed an increasing trend, which may be due to the Cd-induced expression of leaf ABA synthesis genes, which promotes the synthesis of ABA in the plant [55,56] and thus enhances the tolerance of Cd in *M. laxiflora*. Therefore, it is possible that Cd stress may be alleviated by increasing the content of ABA in *M. laxiflora*.

### 3.3. Cd Stress on M. laxiflora Exhibits Genetic Differences

Phytohormones have important roles in regulating plant growth and development and abiotic stresses. Mitogen-activated protein kinase (MAPK) belongs to a class of Ser/Thr protein kinases that are commonly found in plants. MAPK is activated when plants are subjected to abiotic stresses, and active MAPK phosphorylates downstream substrates such as kinases, enzymes, and transcription factors (TFs). Studies have shown that the MAPK cascade pathway plays an important role in regulating plant responses to stresses such as heavy metals, high temperatures, and low temperatures [57]. For example, the MAPK pathway in mulberry (Morus alba) is activated by hormones such as ABA and JA, thereby increasing its Cd tolerance [58]; in *Arabidopsis thaliana*, Cd also rapidly promotes the expression of *AtMPK6*, which reduces oxidative stress and enhances Cd tolerance in *Arabidopsis thaliana* [59]. In this study, with the prolongation of the stress time, the genes involved in the response of *M. laxiflora* showed an increasing trend, and the down-regulated genes were more than the up-regulated genes, so it can be seen that Cd stress affects the gene expression of *M. laxiflora*. The GO functional enrichment analysis found that Cd stress inhibited the down-regulation of photosynthesis-related genes, such as photosynthesis, photosynthesis and light harvesting, photosystem II, and the membrane of chloroplast-like vesicle in *M. laxiflora* seedlings, thus affecting their photosynthesis. KEGG enrichment analysis revealed that the MAPK pathway was significantly up-regulated, and further heat map analysis showed that *RAN1*, *MPK3,* and *ERF1* were significantly up-regulated in the ethylene-responsive pathway, so the MAPK signaling pathway might be activated by ethylene to improve the Cd tolerance of *M. laxiflora.*

## 4. Materials and Methods

### 4.1. Community Surveys and Soil Sampling

Field investigation and research on *M. laxiflora* communities were carried out in Jiangjin, Chongqing, and Yibin, Sichuan Province in the upper reaches of the Yangtze River. According to the specific conditions of the field distribution area of *M. laxiflora*, a 5 m × 5 m shrub sample was set up in the typical distribution area of *M. laxiflora*, and the tree, shrub, and herb species in the sample were recorded, and the accompanying plants were photographed one by one, specimens were collected, and the plant species were identified, and the surface soil of the 0–20 cm layer was taken by the five-point sampling method, with 100 g taken from each point, totaling about 500 g. Finally, the soil was taken from 0 to 20 cm surface layer by five-point sampling method, about 100 g from each point and about 500 g in total.

### 4.2. Determination of Heavy Metal Content in Soil

The soil samples were weighed 0.05 g, and the mixture was dissolved until transparent and clear using a microwave dissolver (Speed Wave SW-4, Berghof) under the HNO_3_-H_2_O_2_-HF dissolution system (HNO_3_:H_2_O_2_:HF = 7:2:1, volume ratio). After the samples were digested, the volume was fixed to 50 mL, and the mass fraction of heavy metal elements (mg/kg) in each sample was determined by an inductively coupled plasma emission spectrometer (ICP-OES 6300, Thermo Fisher, Waltham, MA, USA).

### 4.3. Evaluation of Soil Heavy Metal Pollution

The study showed that the sediments in the upper Yangtze River section from Yibin to the Three Gorges Reservoir area had relatively high contamination levels of Cd, Zn, Cu, and Pb [9,11]. Therefore, we chose to detect Cu, Pb, Cd, and Zn in the soils, and to determine the mass fraction of each heavy metal element in each sample by Inductively Coupled Plasma Emission Spectrometry (ICP-OES6300, Thermo Fisher, Waltham, MA, USA). The ground cumulative index (GCI) method was used to assess the heavy metal pollution status of the Sparse Water Cypress population [60]. The formula for calculating the geo-cumulative index is $I_{geo}$ = $\log_{2}C_{n}/(kB_{n})$, where Igeo is the geological cumulative index; Cn is the measured concentration of element n, mg/kg; Bn is the soil background value of element n; and k = 1.5 is a correction factor to take into account the possible variation in the background value caused by diagenesis. The grading criteria for the ground cumulative index are shown in Table S1. Sampling locations are shown in Table S2 (Figure S3).

### 4.4. Seedling Preparation and Experimental Conditions

Collect well-growing *M. laxiflora*, and cut the spikes into 15 cm-long cuttings of 10 cm × 10 cm, with normal water and fertilizer management during the period. Seedlings with uniform growth conditions were implanted into square pots with a diameter of 14 cm and a depth of 10 cm, with trays under the pots. Two plants were transplanted in each pot with 0.5 kg of substrate soil (peat soil:perlite:vermiculite = 3:1:1), and cultivated in an RDN-type artificial climatic chamber. The plants were watered every 5 days, using a first grade of water in the early stage of transplanting, and watered with a 10-fold dilution of the MS nutrient solution after the growth of new leaves. Weeds were cleaned up on a regular basis. In order to more closely match the actual situation of the growth of *M. laxiflora*, soil cultivation was used to simulate the heavy metal stress experiment of *M. laxiflora*, and a total of six treatment concentrations were set up, namely: 0 mg/kg, 0.5 mg/kg, 2 mg/kg, 5 mg/kg, 10 mg/kg, and 15 mg/kg, respectively. According to the required weight of the soil, the corresponding stress concentration of the solution was prepared with CdCl_2_·2.5H_2_O. The solution was uniformly sprayed one layer at a time into the culture substrate, mixed well, and then loaded into pots after aging for 60 d. Each pot was filled with 500 g of soil [61]. Afterwards, *M. laxiflora* cuttings with good and basically uniform growth conditions were selected for treatment, with 3 pots per treatment and 2 cuttings per pot. The MS nutrient solution was sprayed every 5 d during the experiment. The nutrient solution did not leak out from the bottom of the pots, and the indicators were measured and sampled after the experiment was carried out for 90 d. The cuttings were then treated with the MS nutrient solution.

### 4.5. Determination of Heavy Metal Content in Plants

Weighing 0.05 g of plant samples, microwave digestion was carried out using a microwave digestion system (HNO_3_:H_2_O_2_ = 6:2, volume ratio) with HNO_3_-H_2_O_2_ until the liquid was transparent and clarified. After the samples were digested, the volume was fixed to 50 mL, and the mass fraction of heavy metal elements (mg/kg) in each sample was determined by an inductively coupled plasma emission spectrometer (ICP-OES 6300, Thermo Fisher, Waltham, MA, USA).

### 4.6. Measurement of Growth Indicators

Plant height, root length, and fresh weight are the basic parameters to measure the growth status of the plant, in addition to the sparse flowering water hyacinth is a dwarf shrub. The number of branches and branchlets can also reflect the growth status of the plant to a certain extent, so this paper chooses the plant height, root length, and fresh weight, as well as the number of first-order and second-order branches as the morphology indexes [62,63].

### 4.7. Preparation of Leaf Paraffin Sections

Intact leaves were selected and immediately put into the fixative (formaldehyde:glacial acetic acid:70% alcohol = 5:5:90) for fixation, and the ratio of the material to the fixative was 1:20. The methods of material handling and filming were based on the Plant Microscopy Techniques.

### 4.8. Measurement of Photosynthetic Physiological Indicators

Gas exchange parameters were determined and fluorescence parameters were measured using a portable photosynthesizer Li-6400 (Li-6400, Li-Cor, Lincoln, NE, USA) [64,65]. The determination of chlorophyll content was also carried out [66].

### 4.9. Measurement of Physiological and Biochemical Indicators

The activity of antioxidant enzymes in the examined *M. laxiflora* was tested using commercial kits specifically designed for physiological and biochemical indices according to the instructions provided. MDA levels were measured using commercial kits according to Hacer et al. [67]. Pro contents were processed using commercially available assay kits according to the manufacturer’s guidelines and evaluated by enzyme marker at 520 nm UV.

### 4.10. Transcriptome Sequencing and Analysis

Hydroponics was used for transcriptome experiments because it is easier to control the heavy metal concentration and the heavy metal distribution is more uniform. In total, 150 µmol/L CdCl_2_ solution was prepared with CdCl_2_·5H_2_O, and cuttings adapted to hydroponics for one week ahead of time were inserted into culture bottles containing 150 µmol/L CdCl_2_ solution, with two seedlings in each bottle and a total of 20 treatment bottles. Samples were taken after 0 h (control), 24 h, 48 h, and 72 h of treatment, and then quickly put into freezing tubes and frozen with liquid nitrogen. Three replicates were taken at each treatment time point, and the samples were sent to Wuhan Huada Genetics for testing, and the remainder was used for the measurement of the Cd content in the plants.

### 4.11. Bioinformatics Analysis

Filtered Clean reads were obtained from UW Genetics before subsequent analysis of biological information. Intergroup difference analysis was performed using PossionDis under the conditions of Fold Change ≥ 2 and FDR ≤ 0.001, and the differentially expressed gene heatmaps were drawn using the pheatmap function for the differential gene sets. Differential genes were functionally classified according to GO and KEGG annotation results as well as official classifications, and KEGG enrichment analysis was performed using phyper in R software (R 3.6.1). GO enrichment analysis was performed using the TermFinder package (https://metacpan.org/pod/GO::TermFinder, accessed on 2 October 2024). Qvalue ≤ 0.05 was used as the threshold, and those satisfying this condition were defined as significantly enriched in candidate genes. The obtained gene data were uploaded to NCBI and the citation number obtained was PRJNA1164124.

### 4.12. Statistical Analysis

Data were processed using Excel 2016 and SPSS 18.0, and charts were produced using Excel 2016 and OriginPro 2022.

## 5. Conclusions

The plant was subjected to heavy metal Cd stress, and it was observed that the plant’s growth and photosynthesis were inhibited with the increase in heavy metal Cd, and responded to the stress through antioxidant enzymes, osmoregulation, and the endogenous hormone ABA. In addition, comparative transcriptome analyses showed GO enrichment and KEGG enrichment in response to heavy metal Cd stress and revealed that ethylene may activate the MAPK signaling pathway, which enhances the tolerance of Cd to heavy metals in *M. laxiflora*. This study demonstrated the response to Cd stress and revealed the possible key response pathways, which can provide a reference basis for further research on the key response pathways, and help the conservation and research on the endangered plants in the same habitats as *M. laxiflora*.

## Figures and Tables

**Table 1:** Soil heavy metal content of wild populations of *M. laxiflora*.

Population	Cu mg/kg	Zn mg/kg	Cd mg/kg	Pd mg/kg
Sishui village	23.19	129.34	0.65	54.84
Mabianba	21.03	118.18	0.67	47.55
Yinjiawan	12.88	53.48	0.20	15.16
Neizhongba	45.22	105.87	0.28	57.21
Chenghuadao	26.97	119.30	0.33	35.87
Laowuji	32.49	127.47	0.39	36.69
Goupayan	40.53	135.79	0.62	48.73
Mean	28.90 ± 11.32	112.78 ± 27.85	0.45 ± 0.19	42.29 ± 14.48
Soil Background of China	22.6	74.2	0.09	26
Soil Background	28.05	78.16	0.11	27.39

Note: The soil background value is the average of the soil element background value of the Three Gorges Reservoir Area and the soil element background value of Sichuan Province.

**Table 2:** Effect of Cd stress on plant height, root length, and fresh weight of seedlings of *M. laxiflora*.

Concentration of Cd (mg/kg)	Plant Height (cm)	Root Length (cm)	Fresh Weight (g)
0 mg/kg	17.13 ± 0.81 a	17.23 ± 0.64 a	6.36 ± 0.69 ab
0.5 mg/kg	17.35 ± 0.17 a	17.10 ± 0.64 a	6.57 ± 0.41 a
2 mg/kg	17.88 ± 0.57 a	17.05 ± 0.27 a	5.49 ± 0.40 abc
5 mg/kg	14.25 ± 0.38 b	15.30 ± 0.42 a	5.13 ± 0.40 abc
10 mg/kg	10.83 ± 0.40 c	11.15 ± 0.50 b	4.48 ± 0.53 bc
15 mg/kg	11.78 ± 0.38 c	11.03 ± 0.42 b	4.01 ± 0.32 c

Note: The values in the table are expressed as mean ± standard error. Different letters indicate significant differences in indicators between different treatments (*p* < 0.05)

**Table 3:** Effect of Cd stress on the net increase in primary and secondary branches of *M. laxiflora*.

Branches	0 mg/kg	0.5 mg/kg	2 mg/kg	5 mg/kg	10 mg/kg	15 mg/kg
Primary branch	9 ± 0.65 a	8 ± 0.20 a	9 ± 0.37 a	8 ± 0.4 a	8 ± 0.33 a	7 ± 1.20 a
Secondary branches	39 ± 0.75 a	33 ± 1.23 b	27 ± 1.12 c	19 ± 1.06 d	10 ± 1.03 e	12 ± 0.96 e

Note: Different letters indicate significant differences in indicators between different treatments (*p* < 0.05).

## Data Availability

The original contributions presented in the study are included in the article/Supplementary Material, further inquiries can be directed to the corresponding author.

## Supplementary Materials

The following supporting information can be downloaded at: https://www.mdpi.com/article/10.3390/plants13233433/s1.
