# Supplementary material for: Transcriptome Proffling, Physiological and Biochemical Analyses Reveal Comprehensive Insights into Cadmium Stress in Myricaria laxiflora
Source: Plants (Basel). 2024 Dec 6;13(23):3433. doi: 10.3390/plants13233433 (PMC11644049; doi:10.3390/plants13233433)
Supplement: Supplementary file 1 [file plants-13-03433-s001.zip › plants-3299677-supplementary.pdf]

Article

Transcriptome Proffling, Physiological and Biochemical Analyses Reveal Comprehensive Insights Into Cadmium Stress in *Myricaria laxiflora*

Yang Peng [1,†], Yu-Bing Yang [1,†], Jing-Cheng Wang [1], Mao-Yuan Tian [2], Xing-Hai Yuan [2], Zhi-Jiang Yang [1], You-Wei Zuo [1] and Hong-Ping Deng [1,*]

[1] Key Laboratory of Eco-Environment in the Three Gorges Reservoir Region, Ministry of Education, School of Life Sciences, Southwest University, Beibei, Chongqing 400715, China; 15182375576@139.com (Y.P.); yubing_yang2022@163.com (Y.-B.Y.); m13224042763@163.com (J.-C.W.); yzj18337179126@163.com (Z.-J.Y.); youweiz@swu.edu.cn (Y.-W.Z.)

[2] Jiangjin Forestry Bureau, Chongqing 402260, China; 13883396399@163.com (M.-Y.T.); jjlyyxh_2020@163.com (X.-H.Y.)

* Correspondence: denghp@swu.edu.cn; Tel.: +86-13883395687

† These authors contributed equally to this work.

Methods

Primary and secondary branches. The number of primary and secondary branches of the seedlings was determined on the day of stress treatment and at 90 d after treatment, respectively.

Plant height. The height of the plant was determined on the 90th day of the treatment, the length of the cuttings from the top of the main stem to the highest point, measured with a steel tape measure, accurate to 0.01 cm;

Root length. Root length was determined at 90 d of the treatment and measured from the base of the cuttings to the apex of the longest root, using a steel tape measure with an accuracy of 0.01 cm.

Fresh weight. Fresh weight was determined at 90 d of the treatment by weighing the whole seedlings with an electronic balance, accurate to 0.001 g. The weight of the cuttings was determined by using an electronic balance.

Determination of heavy metal content. 0.05 g of plant samples were weighed and subjected to microwave digestion using a microwave digestion system (HNO3:H2O2 =6:2, v/v) until the liquid was clear and transparent. After digestion of the samples, the volume was fixed to 50 ml and the mass fraction (mg/kg) of heavy metal elements in each sample was determined by inductively coupled plasma emission spectrometer (ICP-OES 6300).

Measurement of endogenous hormone indicators. Determination of gibberellin, abscisic acid, and growth hormone content using enzyme-linked adsorption immunoassay (ELISA) (Ping Liu and Mingjun Li, 2016).

Changes in Cd content of thinned water hyacinth branches under Cd stress. The experiment was carried out in an RDN-type artificial climate chamber, referring to the experimental treatments of the same family of plants, tamarisk (Gao et al., 2021), 150 μmol/L CdCl2 solution was prepared with CdCl22.5H2O, and cuttings adapted to hydroponics for one week ahead of time were inserted into culture bottles containing 150 μmol/L CdCl2 solution, with two seedlings in each bottle, for a total of 20 treatments. Bottles. Samples were taken after 0 h (control), 24 h, 48 h and 72 h of treatment, and then quickly put into freezing tubes and frozen with liquid nitrogen. Three replicates were taken at each treatment time point, and the samples were sent to Wuhan Huada Genetics for testing, and the remainder was used for the measurement of the Cd content in the plants.

Table S1. Evaluation Criteria of the Geoacumulation Index.

Level	Geoacumulation index	Pollution level
0	$I_{geo} \leq 0$	Non-polluting
1	$0 < I_{geo} \leq 1$	Light pollution
2	$1 < I_{geo} \leq 2$	Moderately polluted
3	$2 < I_{geo} \leq 3$	Moderate pollution
4	$3 < I_{geo} \leq 4$	Heavier pollutant
5	$4 < I_{geo} \leq 5$	Heavy pollution
6	$5 < I_{geo} \leq 10$	Extreme pollution

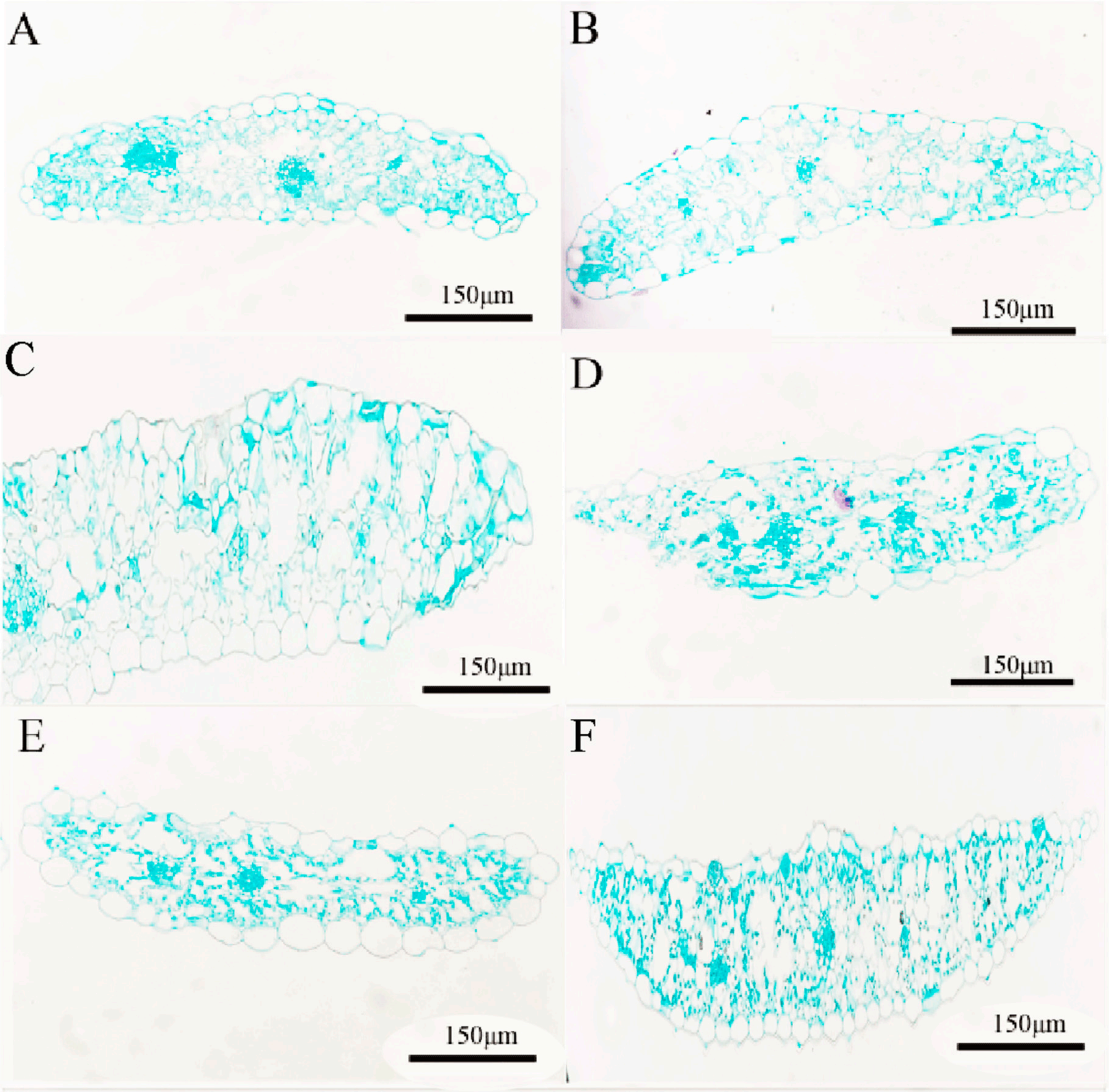

Figure S1. Anatomical Structure of Leaves in Seedlings of *M. laxiflora* under Cd Stress. Note:A-F correspond to 0 mg/kg, 0.5 mg/kg, 2 mg/kg, 5 mg/kg, 10 mg/kg and 15 mg/kg of Cd treated *M. laxiflora* leaves, respectively.

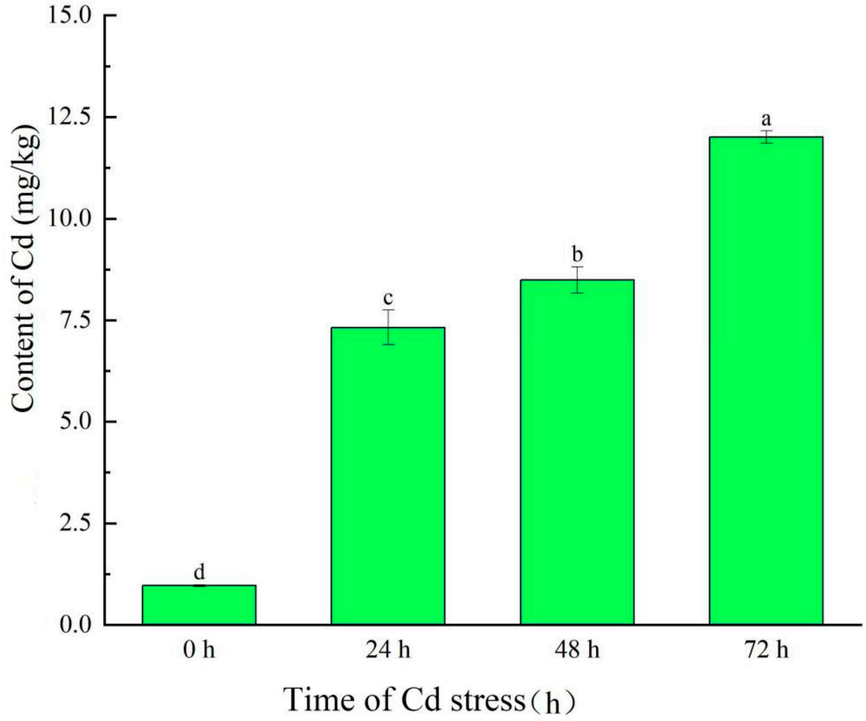

Figure S2. Changes in Cd Content of *M. laxiflora* under Cd Stress. Note: Different letters indicate significant differences in indicators between different treatments（P<0.05）.

Table S2. Information on the Distribution of Wild Populations of *M. laxiflora.*

Region	Number	Population	Longitude/°	Latitude/°	Altitude/m
Jiangjin District, Chongqing	GPY	Goupayan	29.160551	106.125164	192
Jiangjin District, Chongqing	LWJ	Laowuji	29.157934	106.120414	185
Yibin Xuzhou district	MBB	Mabianba	28.971448	104.282871	288
Yibin Xuzhou district	NZB	Neizhongba	28.931299	104.27274	285
Yibin Xuzhou district	SSC	Sishui village	28.898899	104.276751	282
Yibin Nanxi prefecture level city	YJW	Yinjiawan	28.808912	105.034422	249
Yibin Jiang'an county	CHD	Chenghuadao	28.764817	105.029711	246

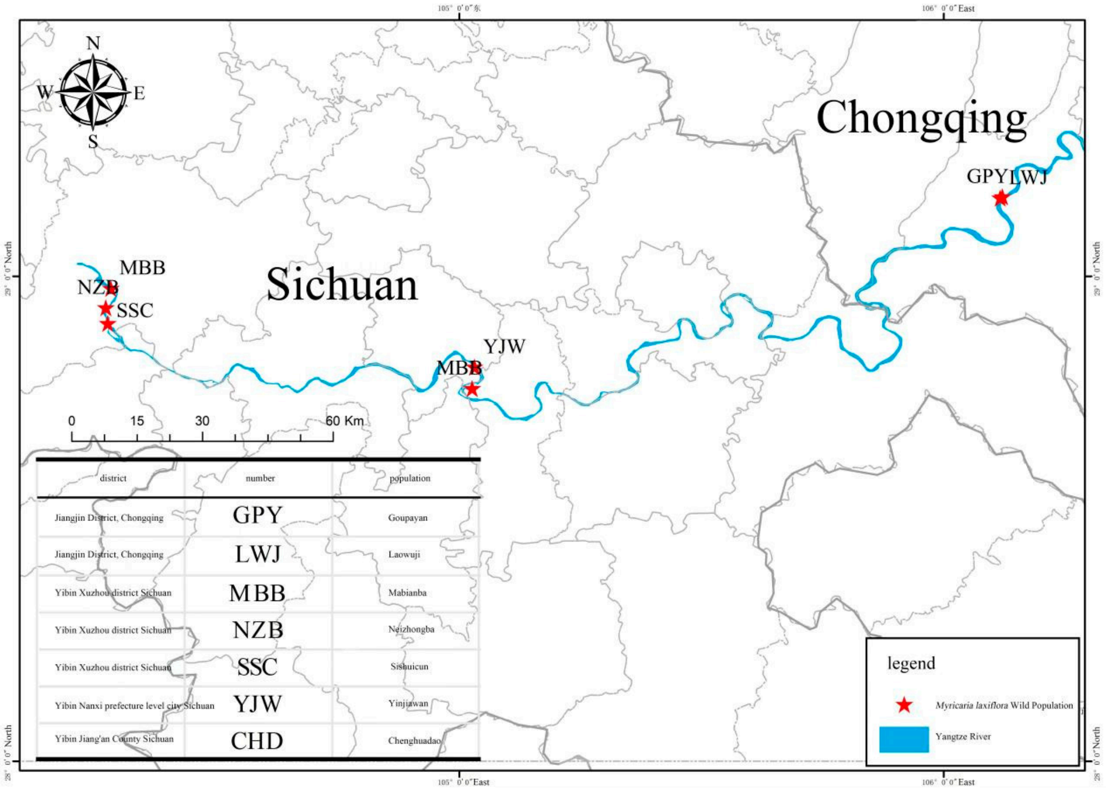

Figure S3. Location of *M. laxiflora* Wild Populations.
